# Octa­butyl­bis­(μ_2_-2-chloro-5-nitro­benzoato)bis­(2-chloro-5-nitro­benzoato)di-μ_3_-oxido-tetra­tin(IV)

**DOI:** 10.1107/S1600536810049317

**Published:** 2010-11-30

**Authors:** Yip-Foo Win, Chen-Shang Choong, Sie-Tiong Ha, Jia Hao Goh, Hoong-Kun Fun

**Affiliations:** a Department of Chemical Science, Faculty of Science, Universiti Tunku Abdul Rahman, 31900 Kampar, Perak, Malaysia; b X-ray Crystallography Unit, School of Physics, Universiti Sains Malaysia, 11800 USM, Penang, Malaysia

## Abstract

The title complex, [Sn_4_(C_4_H_9_)_8_(C_7_H_3_ClNO_4_)_4_O_2_], is a cluster formed by a crystallographic inversion center around the central Sn_2_O_2_ ring. Both of the two independent Sn atoms are five-coordinated, with distorted trigonal–bipyramidal SnC_2_O_3_ geometries. One Sn atom is coordinated by two butyl groups, one O atom of the benzoate anion and two bridging O atoms, whereas the other Sn atom is coordinated by two butyl groups, two O atoms of the benzoate anions and a bridging O atom. The O atoms of the bridging benzoate anion are disordered over two sites with an occupancy ratio of 0.862 (12):0.138 (12). One of the butyl groups coordinated to the Sn_2_O_2_ ring is disordered over two sites with an occupancy ratio of 0.780 (8):0.220 (8), whereas both of the two butyl groups coordinated to the other Sn atom are disordered over two sites with occupancy ratios of 0.788 (5):0.212 (5) and 0.827 (10):0.173 (10). All the butyl groups are equatorial with respect to the SnO_3_ trigonal plane. In the crystal, complex mol­ecules are stacked down [010] with weak inter­molecular C—H⋯π inter­actions stabilizing the crystal structure.

## Related literature

For general background to and applications of the title complex, see: Li *et al.* (2006) ▶; Win *et al.* (2008*a* ▶,*b* ▶, 2010 ▶). For closely related structures, see: Li *et al.* (2006 ▶); Win *et al.* (2008*a* ▶,**b* ▶;* 2010 ▶). For the stability of the temperature controller used in the data collection, see: Cosier & Glazer (1986 ▶).
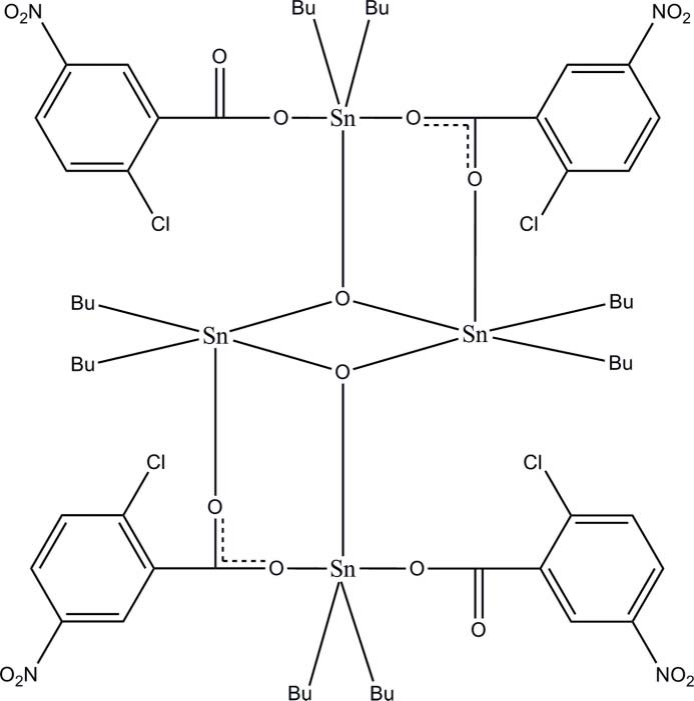


## Experimental

### 

#### Crystal data

[Sn_4_(C_4_H_9_)_8_(C_7_H_3_ClNO_4_)_4_O_2_]
*M* *_r_* = 882.94
Triclinic, 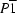

*a* = 13.2278 (14) Å
*b* = 13.2554 (14) Å
*c* = 13.3279 (13) Å
α = 61.523 (2)°
β = 87.345 (2)°
γ = 63.252 (2)°
*V* = 1791.7 (3) Å^3^
*Z* = 2
Mo *K*α radiation
μ = 1.59 mm^−1^
*T* = 100 K
0.27 × 0.17 × 0.09 mm

#### Data collection

Bruker APEXII DUO CCD area-detector diffractometer
Absorption correction: multi-scan (*SADABS*; Bruker, 2009 ▶) *T*_min_ = 0.673, *T*_max_ = 0.867
21637 measured reflections
7668 independent reflections
6167 reflections with *I* > 2σ(*I*)
*R*_int_ = 0.041

#### Refinement

*R*[*F* ^2^ > 2σ(*F* ^2^)] = 0.031
*wR*(*F* ^2^) = 0.078
*S* = 1.04
7668 reflections
466 parameters
221 restraints
H-atom parameters constrained
Δρ_max_ = 0.97 e Å^−3^
Δρ_min_ = −0.88 e Å^−3^

### 

Data collection: *APEX2* (Bruker, 2009 ▶); cell refinement: *SAINT* (Bruker, 2009 ▶); data reduction: *SAINT*; program(s) used to solve structure: *SHELXTL* (Sheldrick, 2008 ▶); program(s) used to refine structure: *SHELXTL*; molecular graphics: *SHELXTL*; software used to prepare material for publication: *SHELXTL* and *PLATON* (Spek, 2009 ▶).

## Supplementary Material

Crystal structure: contains datablocks global, I. DOI: 10.1107/S1600536810049317/ng5076sup1.cif

Structure factors: contains datablocks I. DOI: 10.1107/S1600536810049317/ng5076Isup2.hkl

Additional supplementary materials:  crystallographic information; 3D view; checkCIF report

## Figures and Tables

**Table 1:** Hydrogen-bond geometry (Å, °) *Cg*1 is the centroid of the C16–C21 ring.

*D*—H⋯*A*	*D*—H	H⋯*A*	*D*⋯*A*	*D*—H⋯*A*
C13—H13*A*⋯*Cg*1^i^	0.97	2.92	3.800 (14)	151

Symmetry code: (i) 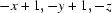.

**Fig. 1.**
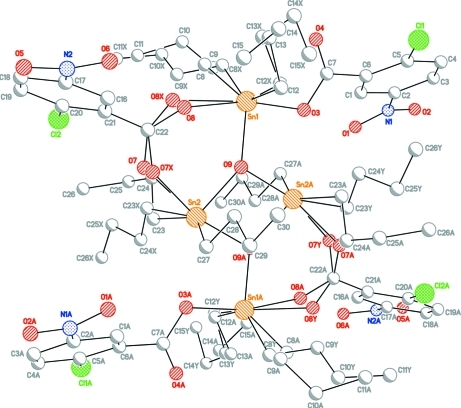
The molecular structure of the title complex, showing the atom-numbering scheme. The suffix A corresponds to symmetry code [-x, -y+1, -z] and minor disordered components are labelled as suffixes X and Y. All H atoms have been omitted for clarity.

**Fig. 2.**
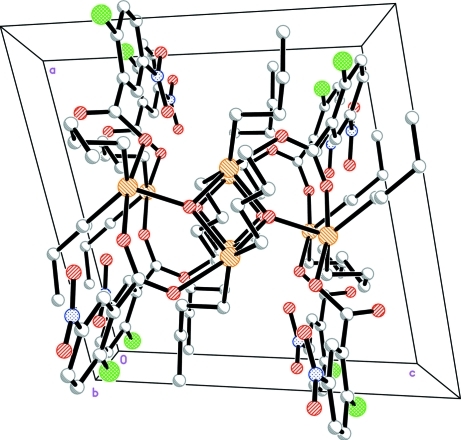
The crystal structure of the title complex, viewed along the b axis, showing the title complex being stacked down the b axis. H atoms have been omitted for clarity.

## supplementary crystallographic information

### Comment

There are many well-documented organodistannoxane dimer complexes with the core geometry consisting of a centrosymmetric planar Sn_2_O_2_ group (Win *et al.*, 2008*a*,*b*,2010). The Sn_2_O_2_ group is bonded to the exo- and endocyclic tin(IV) atom moiety *via* the bridging oxygen atoms resulting the oxygen atoms are tri-coordinated (Li *et al.*, 2006). In this study, the crystal structure of the title complex is similar to the reported organodistannoxane dimer complexes. The only exception is 2-chloro-5-nitrobenzoic acid is utilized in the synthesis to obtain the title complex.

The asymmetric unit of the title complex (Fig. 1) resides on a crystallographic inversion center and comprises of one-half molecule, with the other half of the molecule is generated by symmetry code -x+1, -y, -z+1. Both Sn atoms are five-coordinated in distorted trigonal bipiramidal geometries but the coordination environment is different. The Sn1 atom is coordinated by two butyl groups in equatorial position, an O atom of the monodentate benzoate anion, an O atom of the bridging benzoate anion and a bridging O atom whereas the Sn2 atom is coordinated by two butyl groups in equatorial position, an O atom of the bridging benzoate anion and two bridging O atoms. Atoms O7 and O8 of the bridging benzoate anion are disordered over two sites with occupancy ratio of 0.862 (12):0.138 (12). The two butyl groups (C8-C11 and C12-C15) bonded to the Sn1 atom are disordered over two sites with occupancy ratios of 0.780 (8):0.220 (8) and 0.827 (10):0.173 (10), respectively. At Sn_2_O_2_ ring, one of the butyl group (C23-C26) is disordered over two sites with occupancy ratio of 0.788 (5):0.212 (5). The dihedral angle between the two crystallographically independepent phenyl rings being 25.02 (18)°.

There is no significant intermolecular hydrogen bond observed and the complex are stacked down the [010] axis (Fig. 2). The crystal structure is stabilized by weak intermolecular C13—H13A···*Cg*1 interactions (Table 1) involving the C16-C21 phenyl ring.

### Experimental

The title complex was obtained by heating under reflux a 1:1 molar mixture of dibutyltin(IV) oxide (0.25 g, 1 mmol) and 2-chloro-5-nitrobenzoic acid (0.21 g, 1 mmol) in ethanol (50 ml) for 4 h. Clear solution was isolated by filtration and kept in a bottle. After a few days, colourless single crystals (0.27 g, 61.3 % yield) were collected. *M.p.* 429.2–431.4 K. Analysis found for C_60_H_84_N_4_O_18_Cl_4_Sn_4_: C, 40.89; H, 4.99; N, 3.11 %. Calculated found for C_60_H_84_N_4_O_18_Cl_4_Sn_4_: C, 40.81; H, 4.80; N, 3.17 %.

### Refinement

All H atoms were placed in their calculated positions, with C—H = 0.93 – 0.97 Å, and refined using a riding model with *U*_iso_ = 1.2 or 1.5 *U*_eq_(C). Atoms O7 and O8 were disordered over two sites with a refined occupancy ratio of 0.862 (12):0.138 (12). The butyl groups (C8-C11, C12-C15 and C23-C26) were disordered over two sites with refined occupancy ratios of 0.780 (8):0.220 (8), 0.827 (10):0.173 (10) and 0.788 (5):0.212 (5), respectively. All minor disordered components were refined isotropically. Similarity, simulation and rigid restraints were applied to the disordered components. The C—C distances involving the minor disordered butyl groups were restrained to 1.50 (1) Å. The Sn1—C12X and Sn2—C23X distances were restrained to 2.10 (1) Å. The highest residual electron density peak and the deepest hole were located at 1.02 and 0.71 Å, respectively, from atom Sn1.

### Crystal data

**Table:**

[Sn_4_(C_4_H_9_)_8_(C_7_H_3_ClNO_4_)_4_O_2_]	*Z* = 2
*M**_r_* = 882.94	*F*(000) = 884
Triclinic, *P*1	*D*_x_ = 1.637 Mg m^−^^3^
Hall symbol: -P 1	Mo *K*α radiation, λ = 0.71073 Å
*a* = 13.2278 (14) Å	Cell parameters from 8142 reflections
*b* = 13.2554 (14) Å	θ = 3.0–29.9°
*c* = 13.3279 (13) Å	µ = 1.59 mm^−^^1^
α = 61.523 (2)°	*T* = 100 K
β = 87.345 (2)°	Plate, colourless
γ = 63.252 (2)°	0.27 × 0.17 × 0.09 mm
*V* = 1791.7 (3) Å^3^	

### Data collection

**Table:**

Bruker APEXII DUO CCD area-detector diffractometer	7668 independent reflections
Radiation source: fine-focus sealed tube	6167 reflections with *I* > 2σ(*I*)
graphite	*R*_int_ = 0.041
φ and ω scans	θ_max_ = 27.0°, θ_min_ = 1.9°
Absorption correction: multi-scan (*SADABS*; Bruker, 2009)	*h* = −15→16
*T*_min_ = 0.673, *T*_max_ = 0.867	*k* = −16→16
21637 measured reflections	*l* = −17→16

### Refinement

**Table:**

Refinement on *F*^2^	Primary atom site location: structure-invariant direct methods
Least-squares matrix: full	Secondary atom site location: difference Fourier map
*R*[*F*^2^ > 2σ(*F*^2^)] = 0.031	Hydrogen site location: inferred from neighbouring sites
*wR*(*F*^2^) = 0.078	H-atom parameters constrained
*S* = 1.04	*w* = 1/[σ^2^(*F*_o_^2^) + (0.0196*P*)^2^ + 2.5482*P*] where *P* = (*F*_o_^2^ + 2*F*_c_^2^)/3
7668 reflections	(Δ/σ)_max_ = 0.001
466 parameters	Δρ_max_ = 0.97 e Å^−^^3^
221 restraints	Δρ_min_ = −0.88 e Å^−^^3^

### Special details

**Table:**

Experimental. The crystal was placed in the cold stream of an Oxford Cryosystems Cobra open-flow nitrogen cryostat (Cosier & Glazer, 1986) operating at 100.0 (1)K.
Geometry. All esds (except the esd in the dihedral angle between two l.s. planes) are estimated using the full covariance matrix. The cell esds are taken into account individually in the estimation of esds in distances, angles and torsion angles; correlations between esds in cell parameters are only used when they are defined by crystal symmetry. An approximate (isotropic) treatment of cell esds is used for estimating esds involving l.s. planes.
Refinement. Refinement of F^2^ against ALL reflections. The weighted R-factor wR and goodness of fit S are based on F^2^, conventional R-factors R are based on F, with F set to zero for negative F^2^. The threshold expression of F^2^ > 2sigma(F^2^) is used only for calculating R-factors(gt) etc. and is not relevant to the choice of reflections for refinement. R-factors based on F^2^ are statistically about twice as large as those based on F, and R- factors based on ALL data will be even larger.

### Fractional atomic coordinates and isotropic or equivalent isotropic displacement parameters (Å^2^)

**Table:**

	*x*	*y*	*z*	*U*_iso_*/*U*_eq_	Occ. (<1)
Sn1	0.56558 (2)	0.15969 (2)	0.249637 (19)	0.02618 (7)	
Sn2	0.380257 (19)	0.13444 (2)	0.475501 (18)	0.02152 (7)	
Cl1	1.02684 (11)	−0.10588 (11)	0.27696 (10)	0.0521 (3)	
Cl2	0.08388 (10)	0.50609 (10)	0.10191 (9)	0.0484 (3)	
O1	0.7938 (3)	−0.4845 (3)	0.3840 (3)	0.0674 (10)	
O2	0.9722 (4)	−0.6211 (3)	0.4016 (3)	0.0700 (11)	
O3	0.6949 (2)	−0.0451 (2)	0.3168 (2)	0.0304 (5)	
O4	0.7651 (3)	0.0447 (3)	0.1638 (2)	0.0439 (7)	
O5	0.1796 (3)	0.9708 (3)	0.0553 (3)	0.0538 (8)	
O6	0.3583 (3)	0.8132 (3)	0.1108 (3)	0.0505 (8)	
O7	0.2895 (3)	0.3404 (3)	0.3116 (3)	0.0337 (11)	0.862 (12)
O8	0.4278 (4)	0.3625 (3)	0.2169 (4)	0.0461 (16)	0.862 (12)
O7X	0.319 (2)	0.355 (2)	0.3350 (18)	0.024 (5)*	0.138 (12)
O8X	0.3919 (18)	0.3494 (17)	0.1808 (15)	0.021 (5)*	0.138 (12)
O9	0.5135 (2)	0.0726 (2)	0.39818 (18)	0.0228 (5)	
N1	0.8976 (4)	−0.5134 (3)	0.3783 (3)	0.0478 (9)	
N2	0.2552 (4)	0.8570 (3)	0.0834 (3)	0.0394 (8)	
C1	0.8404 (4)	−0.2853 (4)	0.3168 (3)	0.0341 (8)	
H1A	0.7637	−0.2674	0.3132	0.041*	
C2	0.9292 (4)	−0.4106 (4)	0.3482 (3)	0.0350 (9)	
C3	1.0435 (4)	−0.4424 (4)	0.3558 (3)	0.0427 (10)	
H3A	1.1006	−0.5268	0.3761	0.051*	
C4	1.0722 (4)	−0.3467 (4)	0.3326 (3)	0.0418 (10)	
H4A	1.1494	−0.3665	0.3381	0.050*	
C5	0.9857 (4)	−0.2208 (4)	0.3009 (3)	0.0362 (9)	
C6	0.8683 (3)	−0.1871 (4)	0.2908 (3)	0.0311 (8)	
C7	0.7723 (3)	−0.0512 (4)	0.2519 (3)	0.0307 (8)	
C8	0.4617 (6)	0.2056 (7)	0.1038 (4)	0.0455 (15)	0.780 (8)
H8A	0.3824	0.2661	0.0980	0.055*	0.780 (8)
H8B	0.4860	0.2518	0.0345	0.055*	0.780 (8)
C9	0.4623 (7)	0.0949 (6)	0.1016 (6)	0.065 (2)	0.780 (8)
H9A	0.4382	0.0477	0.1706	0.078*	0.780 (8)
H9B	0.5409	0.0347	0.1052	0.078*	0.780 (8)
C10	0.3820 (7)	0.1382 (8)	−0.0081 (6)	0.073 (2)	0.780 (8)
H10A	0.4063	0.1854	−0.0768	0.088*	0.780 (8)
H10B	0.3922	0.0602	−0.0065	0.088*	0.780 (8)
C11	0.2557 (8)	0.2235 (9)	−0.0202 (6)	0.077 (3)	0.780 (8)
H11A	0.2123	0.2458	−0.0902	0.116*	0.780 (8)
H11B	0.2440	0.3024	−0.0242	0.116*	0.780 (8)
H11C	0.2299	0.1769	0.0462	0.116*	0.780 (8)
C8X	0.5005 (15)	0.138 (2)	0.1177 (17)	0.034 (4)*	0.220 (8)
H8XA	0.5270	0.1765	0.0471	0.041*	0.220 (8)
H8XB	0.5337	0.0452	0.1454	0.041*	0.220 (8)
C9X	0.3722 (15)	0.200 (2)	0.088 (2)	0.065 (5)*	0.220 (8)
H9XA	0.3400	0.2942	0.0551	0.078*	0.220 (8)
H9XB	0.3458	0.1676	0.1605	0.078*	0.220 (8)
C10X	0.323 (2)	0.179 (4)	0.005 (3)	0.076 (7)*	0.220 (8)
H10C	0.3571	0.2011	−0.0630	0.091*	0.220 (8)
H10D	0.3474	0.0859	0.0426	0.091*	0.220 (8)
C11X	0.194 (2)	0.253 (4)	−0.037 (3)	0.097 (8)*	0.220 (8)
H11D	0.1732	0.2308	−0.0889	0.145*	0.220 (8)
H11E	0.1674	0.3460	−0.0783	0.145*	0.220 (8)
H11F	0.1576	0.2310	0.0284	0.145*	0.220 (8)
C12	0.6759 (16)	0.2280 (18)	0.2698 (10)	0.0438 (16)	0.827 (10)
H12A	0.6508	0.2619	0.3220	0.053*	0.827 (10)
H12B	0.7541	0.1548	0.3054	0.053*	0.827 (10)
C13	0.6765 (10)	0.3368 (12)	0.1525 (10)	0.0616 (19)	0.827 (10)
H13A	0.7265	0.2957	0.1123	0.074*	0.827 (10)
H13B	0.5987	0.3922	0.1044	0.074*	0.827 (10)
C14	0.7162 (7)	0.4228 (7)	0.1618 (8)	0.077 (2)	0.827 (10)
H14A	0.7908	0.3671	0.2158	0.092*	0.827 (10)
H14B	0.7270	0.4765	0.0859	0.092*	0.827 (10)
C15	0.6339 (7)	0.5109 (7)	0.2022 (6)	0.072 (2)	0.827 (10)
H15A	0.6639	0.5636	0.2051	0.108*	0.827 (10)
H15B	0.6252	0.4586	0.2787	0.108*	0.827 (10)
H15C	0.5600	0.5672	0.1488	0.108*	0.827 (10)
C12X	0.675 (9)	0.231 (11)	0.260 (6)	0.062 (7)*	0.173 (10)
H12C	0.6588	0.2567	0.3184	0.074*	0.173 (10)
H12D	0.7551	0.1611	0.2850	0.074*	0.173 (10)
C13X	0.661 (5)	0.347 (7)	0.144 (5)	0.070 (6)*	0.173 (10)
H13C	0.6618	0.3271	0.0832	0.084*	0.173 (10)
H13D	0.5862	0.4224	0.1272	0.084*	0.173 (10)
C14X	0.754 (4)	0.384 (4)	0.142 (3)	0.076 (6)*	0.173 (10)
H14C	0.8245	0.3213	0.1347	0.091*	0.173 (10)
H14D	0.7300	0.4700	0.0747	0.091*	0.173 (10)
C15X	0.779 (4)	0.385 (5)	0.250 (3)	0.097 (8)*	0.173 (10)
H15D	0.8444	0.3990	0.2486	0.145*	0.173 (10)
H15E	0.7966	0.3018	0.3181	0.145*	0.173 (10)
H15F	0.7127	0.4539	0.2532	0.145*	0.173 (10)
C16	0.2873 (3)	0.6362 (3)	0.1496 (3)	0.0286 (8)	
H16A	0.3624	0.6027	0.1862	0.034*	
C17	0.2146 (4)	0.7687 (4)	0.0871 (3)	0.0319 (8)	
C18	0.1025 (4)	0.8243 (4)	0.0280 (3)	0.0373 (9)	
H18A	0.0548	0.9150	−0.0139	0.045*	
C19	0.0640 (4)	0.7415 (4)	0.0330 (3)	0.0373 (9)	
H19A	−0.0101	0.7760	−0.0070	0.045*	
C20	0.1365 (4)	0.6060 (3)	0.0981 (3)	0.0324 (8)	
C21	0.2478 (3)	0.5513 (3)	0.1580 (3)	0.0253 (7)	
C22	0.3273 (3)	0.4057 (3)	0.2329 (3)	0.0267 (7)	
C23	0.2412 (4)	0.1154 (6)	0.4272 (5)	0.0322 (12)	0.788 (5)
H23A	0.2441	0.0361	0.4907	0.039*	0.788 (5)
H23B	0.1685	0.1894	0.4170	0.039*	0.788 (5)
C24	0.2430 (5)	0.1096 (5)	0.3162 (4)	0.0374 (12)	0.788 (5)
H24A	0.3197	0.0438	0.3221	0.045*	0.788 (5)
H24B	0.2291	0.1938	0.2509	0.045*	0.788 (5)
C25	0.1543 (5)	0.0773 (5)	0.2904 (5)	0.0466 (14)	0.788 (5)
H25A	0.1683	0.0628	0.2251	0.056*	0.788 (5)
H25B	0.1639	−0.0035	0.3578	0.056*	0.788 (5)
C26	0.0325 (6)	0.1842 (7)	0.2620 (6)	0.0638 (19)	0.788 (5)
H26A	−0.0203	0.1595	0.2464	0.096*	0.788 (5)
H26B	0.0220	0.2640	0.1943	0.096*	0.788 (5)
H26C	0.0177	0.1976	0.3270	0.096*	0.788 (5)
C23X	0.2346 (19)	0.155 (3)	0.3955 (18)	0.045 (6)*	0.212 (5)
H23C	0.1742	0.2455	0.3642	0.054*	0.212 (5)
H23D	0.2542	0.1439	0.3291	0.054*	0.212 (5)
C24X	0.1815 (18)	0.070 (2)	0.4615 (16)	0.052 (5)*	0.212 (5)
H24C	0.1469	0.0924	0.5187	0.063*	0.212 (5)
H24D	0.2427	−0.0210	0.5039	0.063*	0.212 (5)
C25X	0.0908 (19)	0.081 (2)	0.3867 (17)	0.059 (5)*	0.212 (5)
H25C	0.0362	0.1734	0.3345	0.071*	0.212 (5)
H25D	0.1278	0.0438	0.3391	0.071*	0.212 (5)
C26X	0.026 (3)	0.013 (4)	0.456 (3)	0.097 (10)*	0.212 (5)
H26D	−0.0306	0.0238	0.4041	0.145*	0.212 (5)
H26E	0.0789	−0.0785	0.5069	0.145*	0.212 (5)
H26F	−0.0126	0.0513	0.5021	0.145*	0.212 (5)
C27	0.4218 (4)	0.2140 (4)	0.5623 (3)	0.0337 (8)	
H27A	0.3519	0.2914	0.5507	0.040*	
H27B	0.4461	0.1502	0.6454	0.040*	
C28	0.5164 (4)	0.2521 (4)	0.5239 (3)	0.0358 (9)	
H28A	0.5822	0.1804	0.5216	0.043*	
H28B	0.4869	0.3284	0.4455	0.043*	
C29	0.5561 (4)	0.2830 (5)	0.6057 (4)	0.0501 (11)	
H29A	0.4890	0.3507	0.6114	0.060*	
H29B	0.5890	0.2049	0.6829	0.060*	
C30	0.6441 (5)	0.3286 (6)	0.5683 (5)	0.0690 (16)	
H30A	0.6626	0.3496	0.6222	0.104*	
H30B	0.6129	0.4052	0.4914	0.104*	
H30C	0.7131	0.2600	0.5674	0.104*	

### Atomic displacement parameters (Å^2^)

**Table:**

	*U*^11^	*U*^22^	*U*^33^	*U*^12^	*U*^13^	*U*^23^
Sn1	0.02598 (14)	0.01702 (12)	0.02233 (11)	−0.00603 (10)	0.00790 (9)	−0.00531 (9)
Sn2	0.01735 (12)	0.01475 (11)	0.02208 (11)	−0.00367 (9)	0.00397 (8)	−0.00608 (9)
Cl1	0.0452 (7)	0.0433 (6)	0.0618 (7)	−0.0249 (5)	0.0171 (5)	−0.0200 (5)
Cl2	0.0470 (6)	0.0276 (5)	0.0531 (6)	−0.0130 (5)	−0.0125 (5)	−0.0114 (4)
O1	0.059 (3)	0.046 (2)	0.096 (3)	−0.0268 (19)	0.006 (2)	−0.033 (2)
O2	0.089 (3)	0.0323 (18)	0.083 (2)	−0.0214 (19)	0.030 (2)	−0.0339 (18)
O3	0.0316 (14)	0.0201 (12)	0.0299 (12)	−0.0086 (11)	0.0138 (11)	−0.0105 (10)
O4	0.0492 (19)	0.0256 (14)	0.0357 (14)	−0.0124 (13)	0.0218 (13)	−0.0077 (12)
O5	0.073 (2)	0.0277 (16)	0.0622 (19)	−0.0247 (17)	0.0286 (18)	−0.0247 (15)
O6	0.060 (2)	0.0399 (18)	0.0489 (17)	−0.0293 (17)	0.0091 (16)	−0.0157 (14)
O7	0.0254 (18)	0.0201 (16)	0.0292 (16)	−0.0034 (14)	0.0057 (14)	−0.0015 (13)
O8	0.031 (2)	0.0191 (16)	0.058 (2)	−0.0043 (15)	0.0182 (19)	−0.0062 (15)
O9	0.0209 (12)	0.0162 (11)	0.0206 (10)	−0.0050 (10)	0.0066 (9)	−0.0060 (9)
N1	0.059 (3)	0.0302 (19)	0.049 (2)	−0.0149 (19)	0.0126 (19)	−0.0232 (17)
N2	0.054 (2)	0.0283 (18)	0.0324 (16)	−0.0211 (18)	0.0166 (16)	−0.0131 (14)
C1	0.037 (2)	0.0272 (19)	0.0284 (17)	−0.0098 (17)	0.0084 (16)	−0.0135 (15)
C2	0.039 (2)	0.0267 (19)	0.0314 (18)	−0.0096 (18)	0.0121 (17)	−0.0160 (16)
C3	0.047 (3)	0.027 (2)	0.0331 (19)	−0.0051 (19)	0.0111 (18)	−0.0123 (17)
C4	0.035 (2)	0.037 (2)	0.036 (2)	−0.0111 (19)	0.0154 (18)	−0.0134 (18)
C5	0.035 (2)	0.030 (2)	0.0330 (18)	−0.0126 (18)	0.0162 (17)	−0.0132 (16)
C6	0.038 (2)	0.0242 (18)	0.0228 (16)	−0.0112 (17)	0.0133 (15)	−0.0101 (14)
C7	0.032 (2)	0.0252 (18)	0.0298 (17)	−0.0116 (16)	0.0127 (15)	−0.0131 (15)
C8	0.039 (3)	0.045 (4)	0.029 (2)	−0.011 (3)	0.002 (2)	−0.011 (3)
C9	0.072 (4)	0.045 (3)	0.057 (3)	−0.015 (3)	−0.011 (3)	−0.023 (3)
C10	0.078 (6)	0.072 (5)	0.063 (4)	−0.022 (4)	−0.008 (4)	−0.041 (4)
C11	0.080 (6)	0.087 (6)	0.049 (4)	−0.051 (5)	−0.015 (4)	−0.011 (4)
C12	0.055 (3)	0.046 (3)	0.052 (4)	−0.038 (3)	0.028 (3)	−0.030 (3)
C13	0.092 (5)	0.054 (4)	0.062 (4)	−0.050 (4)	0.053 (4)	−0.035 (3)
C14	0.054 (4)	0.048 (4)	0.104 (5)	−0.028 (3)	0.024 (4)	−0.020 (4)
C15	0.106 (6)	0.052 (4)	0.062 (4)	−0.045 (4)	0.019 (4)	−0.025 (3)
C16	0.0274 (19)	0.0237 (18)	0.0250 (16)	−0.0073 (15)	0.0105 (14)	−0.0109 (14)
C17	0.046 (2)	0.0230 (18)	0.0264 (16)	−0.0170 (18)	0.0154 (16)	−0.0126 (15)
C18	0.040 (2)	0.0186 (18)	0.0293 (18)	−0.0042 (17)	0.0025 (16)	−0.0042 (15)
C19	0.033 (2)	0.0222 (18)	0.0326 (18)	−0.0020 (17)	−0.0042 (16)	−0.0067 (16)
C20	0.038 (2)	0.0209 (18)	0.0273 (17)	−0.0082 (16)	0.0013 (16)	−0.0095 (15)
C21	0.0245 (18)	0.0170 (16)	0.0227 (15)	−0.0032 (14)	0.0071 (13)	−0.0087 (13)
C22	0.0243 (19)	0.0186 (16)	0.0302 (17)	−0.0070 (15)	0.0021 (14)	−0.0106 (14)
C23	0.016 (2)	0.034 (3)	0.034 (3)	−0.009 (2)	−0.0011 (19)	−0.011 (2)
C24	0.038 (3)	0.036 (3)	0.037 (2)	−0.018 (2)	0.002 (2)	−0.017 (2)
C25	0.048 (3)	0.040 (3)	0.049 (3)	−0.018 (3)	−0.001 (3)	−0.023 (3)
C26	0.049 (4)	0.062 (4)	0.079 (4)	−0.018 (3)	−0.004 (3)	−0.042 (4)
C27	0.043 (2)	0.0291 (19)	0.0353 (19)	−0.0196 (18)	0.0137 (17)	−0.0191 (16)
C28	0.034 (2)	0.033 (2)	0.045 (2)	−0.0165 (18)	0.0125 (17)	−0.0234 (18)
C29	0.058 (3)	0.054 (3)	0.056 (3)	−0.033 (3)	0.014 (2)	−0.035 (2)
C30	0.065 (4)	0.073 (4)	0.093 (4)	−0.045 (3)	0.011 (3)	−0.049 (3)

### Geometric parameters (Å, °)

**Table:**

Sn1—O9	2.034 (2)	C13—H13A	0.9700
Sn1—C12X	2.096 (10)	C13—H13B	0.9700
Sn1—C8	2.098 (5)	C14—C15	1.497 (10)
Sn1—C12	2.116 (5)	C14—H14A	0.9700
Sn1—O3	2.177 (2)	C14—H14B	0.9700
Sn1—C8X	2.180 (19)	C15—H15A	0.9600
Sn1—O8X	2.289 (19)	C15—H15B	0.9600
Sn1—O8	2.299 (3)	C15—H15C	0.9600
Sn2—O9	2.044 (2)	C12X—C13X	1.513 (9)
Sn2—C23X	2.088 (10)	C12X—H12C	0.9700
Sn2—C27	2.121 (3)	C12X—H12D	0.9700
Sn2—C23	2.131 (4)	C13X—C14X	1.504 (9)
Sn2—O9^i^	2.164 (2)	C13X—H13C	0.9700
Sn2—O7	2.296 (3)	C13X—H13D	0.9700
Sn2—O7X	2.35 (2)	C14X—C15X	1.498 (9)
Cl1—C5	1.728 (4)	C14X—H14C	0.9700
Cl2—C20	1.733 (4)	C14X—H14D	0.9700
O1—N1	1.257 (5)	C15X—H15D	0.9600
O2—N1	1.202 (5)	C15X—H15E	0.9600
O3—C7	1.309 (4)	C15X—H15F	0.9600
O4—C7	1.217 (4)	C16—C17	1.364 (5)
O5—N2	1.246 (5)	C16—C21	1.398 (5)
O6—N2	1.208 (5)	C16—H16A	0.9300
O7—C22	1.255 (5)	C17—C18	1.391 (6)
O8—C22	1.243 (5)	C18—C19	1.378 (6)
O7X—C22	1.22 (2)	C18—H18A	0.9300
O8X—C22	1.279 (19)	C19—C20	1.393 (5)
O9—Sn2^i^	2.164 (2)	C19—H19A	0.9300
N1—C2	1.474 (5)	C20—C21	1.389 (5)
N2—C17	1.472 (5)	C21—C22	1.503 (5)
C1—C2	1.393 (5)	C23—C24	1.517 (7)
C1—C6	1.394 (5)	C23—H23A	0.9700
C1—H1A	0.9300	C23—H23B	0.9700
C2—C3	1.369 (6)	C24—C25	1.519 (7)
C3—C4	1.376 (6)	C24—H24A	0.9700
C3—H3A	0.9300	C24—H24B	0.9700
C4—C5	1.388 (6)	C25—C26	1.495 (8)
C4—H4A	0.9300	C25—H25A	0.9700
C5—C6	1.402 (6)	C25—H25B	0.9700
C6—C7	1.496 (5)	C26—H26A	0.9600
C8—C9	1.478 (8)	C26—H26B	0.9600
C8—H8A	0.9700	C26—H26C	0.9600
C8—H8B	0.9700	C23X—C24X	1.502 (9)
C9—C10	1.546 (8)	C23X—H23C	0.9700
C9—H9A	0.9700	C23X—H23D	0.9700
C9—H9B	0.9700	C24X—C25X	1.505 (9)
C10—C11	1.500 (11)	C24X—H24C	0.9700
C10—H10A	0.9700	C24X—H24D	0.9700
C10—H10B	0.9700	C25X—C26X	1.493 (9)
C11—H11A	0.9600	C25X—H25C	0.9700
C11—H11B	0.9600	C25X—H25D	0.9700
C11—H11C	0.9600	C26X—C26X^ii^	1.31 (5)
C8X—C9X	1.487 (9)	C26X—H26D	0.9601
C8X—H8XA	0.9700	C26X—H26E	0.9600
C8X—H8XB	0.9700	C26X—H26F	0.9600
C9X—C10X	1.512 (9)	C27—C28	1.532 (5)
C9X—H9XA	0.9700	C27—H27A	0.9700
C9X—H9XB	0.9700	C27—H27B	0.9700
C10X—C11X	1.499 (9)	C28—C29	1.518 (5)
C10X—H10C	0.9700	C28—H28A	0.9700
C10X—H10D	0.9700	C28—H28B	0.9700
C11X—H11D	0.9600	C29—C30	1.504 (7)
C11X—H11E	0.9600	C29—H29A	0.9700
C11X—H11F	0.9600	C29—H29B	0.9700
C12—C13	1.543 (7)	C30—H30A	0.9600
C12—H12A	0.9700	C30—H30B	0.9600
C12—H12B	0.9700	C30—H30C	0.9600
C13—C14	1.507 (9)		
			
O9—Sn1—C12X	117 (2)	C14—C13—H13B	108.4
O9—Sn1—C8	110.57 (17)	C12—C13—H13B	108.4
C12X—Sn1—C8	130.4 (18)	H13A—C13—H13B	107.5
O9—Sn1—C12	113.6 (3)	C15—C14—C13	113.9 (7)
C12X—Sn1—C12	3(3)	C15—C14—H14A	108.8
C8—Sn1—C12	133.3 (4)	C13—C14—H14A	108.8
O9—Sn1—O3	80.20 (9)	C15—C14—H14B	108.8
C12X—Sn1—O3	99 (4)	C13—C14—H14B	108.8
C8—Sn1—O3	103.1 (2)	H14A—C14—H14B	107.7
C12—Sn1—O3	98.5 (6)	C14—C15—H15A	109.5
O9—Sn1—C8X	107.7 (5)	C14—C15—H15B	109.5
C12X—Sn1—C8X	135 (2)	H15A—C15—H15B	109.5
C8—Sn1—C8X	19.6 (5)	C14—C15—H15C	109.5
C12—Sn1—C8X	138.4 (6)	H15A—C15—H15C	109.5
O3—Sn1—C8X	83.5 (6)	H15B—C15—H15C	109.5
O9—Sn1—O8X	90.3 (4)	C13X—C12X—Sn1	112 (3)
C12X—Sn1—O8X	99 (4)	C13X—C12X—H12C	109.2
C8—Sn1—O8X	66.0 (6)	Sn1—C12X—H12C	109.2
C12—Sn1—O8X	99.1 (8)	C13X—C12X—H12D	109.2
O3—Sn1—O8X	162.1 (6)	Sn1—C12X—H12D	109.2
C8X—Sn1—O8X	85.0 (8)	H12C—C12X—H12D	107.9
O9—Sn1—O8	89.69 (10)	C14X—C13X—C12X	113 (2)
C12X—Sn1—O8	81 (4)	C14X—C13X—H13C	108.9
C8—Sn1—O8	85.2 (3)	C12X—C13X—H13C	108.9
C12—Sn1—O8	80.7 (6)	C14X—C13X—H13D	108.9
O3—Sn1—O8	168.65 (13)	C12X—C13X—H13D	108.9
C8X—Sn1—O8	104.7 (6)	H13C—C13X—H13D	107.7
O8X—Sn1—O8	20.4 (5)	C15X—C14X—C13X	113 (2)
O9—Sn2—C23X	107.8 (9)	C15X—C14X—H14C	109.0
O9—Sn2—C27	108.23 (13)	C13X—C14X—H14C	109.0
C23X—Sn2—C27	139.5 (10)	C15X—C14X—H14D	109.0
O9—Sn2—C23	109.92 (18)	C13X—C14X—H14D	109.0
C23X—Sn2—C23	12.5 (6)	H14C—C14X—H14D	107.8
C27—Sn2—C23	141.4 (2)	C14X—C15X—H15D	109.5
O9—Sn2—O9^i^	76.18 (9)	C14X—C15X—H15E	109.5
C23X—Sn2—O9^i^	106.8 (7)	H15D—C15X—H15E	109.5
C27—Sn2—O9^i^	99.21 (12)	C14X—C15X—H15F	109.5
C23—Sn2—O9^i^	95.09 (18)	H15D—C15X—H15F	109.5
O9—Sn2—O7	91.26 (10)	H15E—C15X—H15F	109.5
C23X—Sn2—O7	72.3 (6)	C17—C16—C21	119.5 (3)
C27—Sn2—O7	89.01 (18)	C17—C16—H16A	120.3
C23—Sn2—O7	84.7 (2)	C21—C16—H16A	120.3
O9^i^—Sn2—O7	166.62 (11)	C16—C17—C18	122.7 (3)
O9—Sn2—O7X	91.2 (5)	C16—C17—N2	119.0 (4)
C23X—Sn2—O7X	87.7 (9)	C18—C17—N2	118.3 (3)
C27—Sn2—O7X	73.7 (6)	C19—C18—C17	118.3 (3)
C23—Sn2—O7X	99.9 (7)	C19—C18—H18A	120.9
O9^i^—Sn2—O7X	163.1 (6)	C17—C18—H18A	120.9
O7—Sn2—O7X	16.1 (5)	C18—C19—C20	119.7 (4)
C7—O3—Sn1	109.3 (2)	C18—C19—H19A	120.2
C22—O7—Sn2	132.3 (3)	C20—C19—H19A	120.2
C22—O8—Sn1	133.2 (3)	C21—C20—C19	121.7 (3)
C22—O7X—Sn2	130.3 (15)	C21—C20—Cl2	120.4 (3)
C22—O8X—Sn1	131.3 (12)	C19—C20—Cl2	117.9 (3)
Sn1—O9—Sn2	135.82 (11)	C20—C21—C16	118.2 (3)
Sn1—O9—Sn2^i^	120.19 (11)	C20—C21—C22	123.4 (3)
Sn2—O9—Sn2^i^	103.82 (9)	C16—C21—C22	118.3 (3)
O2—N1—O1	123.7 (4)	O7X—C22—O8	115.2 (11)
O2—N1—C2	119.0 (4)	O8—C22—O7	125.7 (3)
O1—N1—C2	117.2 (4)	O7X—C22—O8X	128.2 (13)
O6—N2—O5	125.0 (4)	O7—C22—O8X	116.0 (9)
O6—N2—C17	118.0 (3)	O7X—C22—C21	115.8 (10)
O5—N2—C17	116.9 (4)	O8—C22—C21	116.7 (3)
C2—C1—C6	118.9 (4)	O7—C22—C21	117.3 (3)
C2—C1—H1A	120.6	O8X—C22—C21	115.9 (8)
C6—C1—H1A	120.6	C24—C23—Sn2	114.0 (4)
C3—C2—C1	123.0 (4)	C24—C23—H23A	108.7
C3—C2—N1	119.3 (4)	Sn2—C23—H23A	108.7
C1—C2—N1	117.8 (4)	C24—C23—H23B	108.7
C2—C3—C4	118.6 (4)	Sn2—C23—H23B	108.7
C2—C3—H3A	120.7	H23A—C23—H23B	107.6
C4—C3—H3A	120.7	C23—C24—C25	114.2 (4)
C3—C4—C5	120.0 (4)	C23—C24—H24A	108.7
C3—C4—H4A	120.0	C25—C24—H24A	108.7
C5—C4—H4A	120.0	C23—C24—H24B	108.7
C4—C5—C6	121.7 (4)	C25—C24—H24B	108.7
C4—C5—Cl1	118.0 (3)	H24A—C24—H24B	107.6
C6—C5—Cl1	120.3 (3)	C26—C25—C24	112.4 (5)
C1—C6—C5	117.9 (4)	C26—C25—H25A	109.1
C1—C6—C7	118.7 (4)	C24—C25—H25A	109.1
C5—C6—C7	123.3 (3)	C26—C25—H25B	109.1
O4—C7—O3	123.3 (4)	C24—C25—H25B	109.1
O4—C7—C6	122.0 (3)	H25A—C25—H25B	107.9
O3—C7—C6	114.7 (3)	C25—C26—H26A	109.5
C9—C8—Sn1	117.2 (4)	C25—C26—H26B	109.5
C9—C8—H8A	108.0	H26A—C26—H26B	109.5
Sn1—C8—H8A	108.0	C25—C26—H26C	109.5
C9—C8—H8B	108.0	H26A—C26—H26C	109.5
Sn1—C8—H8B	108.0	H26B—C26—H26C	109.5
H8A—C8—H8B	107.3	C24X—C23X—Sn2	121.6 (12)
C8—C9—C10	113.5 (5)	C24X—C23X—H23C	106.9
C8—C9—H9A	108.9	Sn2—C23X—H23C	106.9
C10—C9—H9A	108.9	C24X—C23X—H23D	106.9
C8—C9—H9B	108.9	Sn2—C23X—H23D	106.9
C10—C9—H9B	108.9	H23C—C23X—H23D	106.7
H9A—C9—H9B	107.7	C23X—C24X—C25X	114.7 (13)
C11—C10—C9	114.8 (7)	C23X—C24X—H24C	108.6
C11—C10—H10A	108.6	C25X—C24X—H24C	108.6
C9—C10—H10A	108.6	C23X—C24X—H24D	108.6
C11—C10—H10B	108.6	C25X—C24X—H24D	108.6
C9—C10—H10B	108.6	H24C—C24X—H24D	107.6
H10A—C10—H10B	107.5	C26X—C25X—C24X	113.1 (17)
C10—C11—H11A	109.5	C26X—C25X—H25C	108.9
C10—C11—H11B	109.5	C24X—C25X—H25C	108.9
H11A—C11—H11B	109.5	C26X—C25X—H25D	108.9
C10—C11—H11C	109.5	C24X—C25X—H25D	108.9
H11A—C11—H11C	109.5	H25C—C25X—H25D	107.8
H11B—C11—H11C	109.5	C26X^ii^—C26X—C25X	149 (4)
C9X—C8X—Sn1	114.2 (13)	C26X^ii^—C26X—H26D	94.5
C9X—C8X—H8XA	108.7	C25X—C26X—H26D	109.4
Sn1—C8X—H8XA	108.7	C26X^ii^—C26X—H26E	79.5
C9X—C8X—H8XB	108.7	C25X—C26X—H26E	109.6
Sn1—C8X—H8XB	108.7	H26D—C26X—H26E	109.5
H8XA—C8X—H8XB	107.6	C25X—C26X—H26F	109.4
C8X—C9X—C10X	116.5 (17)	H26D—C26X—H26F	109.5
C8X—C9X—H9XA	108.2	H26E—C26X—H26F	109.5
C10X—C9X—H9XA	108.2	C28—C27—Sn2	116.0 (3)
C8X—C9X—H9XB	108.2	C28—C27—H27A	108.3
C10X—C9X—H9XB	108.2	Sn2—C27—H27A	108.3
H9XA—C9X—H9XB	107.3	C28—C27—H27B	108.3
C11X—C10X—C9X	118 (2)	Sn2—C27—H27B	108.3
C11X—C10X—H10C	107.9	H27A—C27—H27B	107.4
C9X—C10X—H10C	107.9	C29—C28—C27	111.9 (3)
C11X—C10X—H10D	107.9	C29—C28—H28A	109.2
C9X—C10X—H10D	107.9	C27—C28—H28A	109.2
H10C—C10X—H10D	107.2	C29—C28—H28B	109.2
C10X—C11X—H11D	109.5	C27—C28—H28B	109.2
C10X—C11X—H11E	109.5	H28A—C28—H28B	107.9
H11D—C11X—H11E	109.5	C30—C29—C28	114.2 (4)
C10X—C11X—H11F	109.5	C30—C29—H29A	108.7
H11D—C11X—H11F	109.5	C28—C29—H29A	108.7
H11E—C11X—H11F	109.5	C30—C29—H29B	108.7
C13—C12—Sn1	112.5 (6)	C28—C29—H29B	108.7
C13—C12—H12A	109.1	H29A—C29—H29B	107.6
Sn1—C12—H12A	109.1	C29—C30—H30A	109.5
C13—C12—H12B	109.1	C29—C30—H30B	109.5
Sn1—C12—H12B	109.1	H30A—C30—H30B	109.5
H12A—C12—H12B	107.8	C29—C30—H30C	109.5
C14—C13—C12	115.4 (8)	H30A—C30—H30C	109.5
C14—C13—H13A	108.4	H30B—C30—H30C	109.5
C12—C13—H13A	108.4		
			
O9—Sn1—O3—C7	−173.6 (2)	O9—Sn1—C8X—C9X	60.4 (17)
C12X—Sn1—O3—C7	−58 (2)	C12X—Sn1—C8X—C9X	−126 (5)
C8—Sn1—O3—C7	77.4 (3)	C8—Sn1—C8X—C9X	−41.3 (15)
C12—Sn1—O3—C7	−60.9 (4)	C12—Sn1—C8X—C9X	−126.5 (17)
C8X—Sn1—O3—C7	77.1 (5)	O3—Sn1—C8X—C9X	138.0 (17)
O8X—Sn1—O3—C7	127.5 (15)	O8X—Sn1—C8X—C9X	−28.3 (17)
O8—Sn1—O3—C7	−146.3 (8)	O8—Sn1—C8X—C9X	−34.0 (17)
O9—Sn2—O7—C22	−28.3 (5)	Sn1—C8X—C9X—C10X	−175 (2)
C23X—Sn2—O7—C22	−136.7 (11)	C8X—C9X—C10X—C11X	−173 (3)
C27—Sn2—O7—C22	79.9 (5)	O9—Sn1—C12—C13	−157.9 (10)
C23—Sn2—O7—C22	−138.2 (6)	C8—Sn1—C12—C13	2.0 (18)
O9^i^—Sn2—O7—C22	−48.3 (11)	O3—Sn1—C12—C13	119.1 (12)
O7X—Sn2—O7—C22	62 (2)	C8X—Sn1—C12—C13	29 (2)
O9—Sn1—O8—C22	−36.9 (6)	O8X—Sn1—C12—C13	−63.5 (13)
C12X—Sn1—O8—C22	−154 (2)	O8—Sn1—C12—C13	−72.4 (12)
C8—Sn1—O8—C22	73.7 (6)	Sn1—C12—C13—C14	159.1 (10)
C12—Sn1—O8—C22	−151.0 (7)	C12—C13—C14—C15	−68.8 (13)
O3—Sn1—O8—C22	−63.8 (12)	O9—Sn1—C12X—C13X	−151 (6)
C8X—Sn1—O8—C22	71.3 (8)	C8—Sn1—C12X—C13X	10 (11)
O8X—Sn1—O8—C22	54.9 (13)	O3—Sn1—C12X—C13X	126 (8)
O9—Sn2—O7X—C22	29 (2)	C8X—Sn1—C12X—C13X	36 (11)
C23X—Sn2—O7X—C22	−79 (2)	O8X—Sn1—C12X—C13X	−56 (8)
C27—Sn2—O7X—C22	138 (2)	O8—Sn1—C12X—C13X	−66 (8)
C23—Sn2—O7X—C22	−81 (2)	Sn1—C12X—C13X—C14X	−169 (5)
O9^i^—Sn2—O7X—C22	71 (3)	C12X—C13X—C14X—C15X	−45 (8)
O7—Sn2—O7X—C22	−61 (2)	C21—C16—C17—C18	−1.8 (5)
O9—Sn1—O8X—C22	37.7 (19)	C21—C16—C17—N2	177.2 (3)
C12X—Sn1—O8X—C22	−79 (3)	O6—N2—C17—C16	17.6 (5)
C8—Sn1—O8X—C22	150 (2)	O5—N2—C17—C16	−160.4 (3)
C12—Sn1—O8X—C22	−76.3 (19)	O6—N2—C17—C18	−163.4 (3)
O3—Sn1—O8X—C22	95 (3)	O5—N2—C17—C18	18.7 (5)
C8X—Sn1—O8X—C22	145 (2)	C16—C17—C18—C19	0.0 (6)
O8—Sn1—O8X—C22	−50.5 (15)	N2—C17—C18—C19	−179.0 (3)
C12X—Sn1—O9—Sn2	88 (4)	C17—C18—C19—C20	1.2 (6)
C8—Sn1—O9—Sn2	−76.8 (3)	C18—C19—C20—C21	−0.5 (6)
C12—Sn1—O9—Sn2	87.8 (7)	C18—C19—C20—Cl2	179.8 (3)
O3—Sn1—O9—Sn2	−177.14 (18)	C19—C20—C21—C16	−1.3 (5)
C8X—Sn1—O9—Sn2	−97.3 (6)	Cl2—C20—C21—C16	178.3 (3)
O8X—Sn1—O9—Sn2	−12.4 (6)	C19—C20—C21—C22	177.6 (4)
O8—Sn1—O9—Sn2	8.0 (2)	Cl2—C20—C21—C22	−2.8 (5)
C12X—Sn1—O9—Sn2^i^	−87 (4)	C17—C16—C21—C20	2.4 (5)
C8—Sn1—O9—Sn2^i^	108.9 (2)	C17—C16—C21—C22	−176.6 (3)
C12—Sn1—O9—Sn2^i^	−86.6 (7)	Sn2—O7X—C22—O8	−57 (2)
O3—Sn1—O9—Sn2^i^	8.54 (11)	Sn2—O7X—C22—O7	61.2 (19)
C8X—Sn1—O9—Sn2^i^	88.4 (6)	Sn2—O7X—C22—O8X	−15 (3)
O8X—Sn1—O9—Sn2^i^	173.3 (6)	Sn2—O7X—C22—C21	161.8 (13)
O8—Sn1—O9—Sn2^i^	−166.29 (19)	Sn1—O8—C22—O7X	63.8 (12)
C23X—Sn2—O9—Sn1	81.5 (7)	Sn1—O8—C22—O7	30.4 (7)
C27—Sn2—O9—Sn1	−79.64 (19)	Sn1—O8—C22—O8X	−56.8 (13)
C23—Sn2—O9—Sn1	94.6 (2)	Sn1—O8—C22—C21	−155.4 (4)
O9^i^—Sn2—O9—Sn1	−174.9 (2)	Sn2—O7—C22—O7X	−67 (2)
O7—Sn2—O9—Sn1	9.7 (2)	Sn2—O7—C22—O8	11.7 (7)
O7X—Sn2—O9—Sn1	−6.4 (7)	Sn2—O7—C22—O8X	54.4 (10)
C23X—Sn2—O9—Sn2^i^	−103.5 (7)	Sn2—O7—C22—C21	−162.5 (3)
C27—Sn2—O9—Sn2^i^	95.31 (13)	Sn1—O8X—C22—O7X	−27 (2)
C23—Sn2—O9—Sn2^i^	−90.46 (19)	Sn1—O8X—C22—O8	54.7 (14)
O9^i^—Sn2—O9—Sn2^i^	0.0	Sn1—O8X—C22—O7	−61 (2)
O7—Sn2—O9—Sn2^i^	−175.32 (17)	Sn1—O8X—C22—C21	155.6 (12)
O7X—Sn2—O9—Sn2^i^	168.6 (6)	C20—C21—C22—O7X	−88.0 (15)
C6—C1—C2—C3	0.5 (6)	C16—C21—C22—O7X	91.0 (14)
C6—C1—C2—N1	178.4 (3)	C20—C21—C22—O8	131.5 (5)
O2—N1—C2—C3	−4.7 (6)	C16—C21—C22—O8	−49.6 (5)
O1—N1—C2—C3	171.6 (4)	C20—C21—C22—O7	−53.8 (6)
O2—N1—C2—C1	177.4 (4)	C16—C21—C22—O7	125.2 (4)
O1—N1—C2—C1	−6.4 (5)	C20—C21—C22—O8X	89.3 (13)
C1—C2—C3—C4	0.8 (6)	C16—C21—C22—O8X	−91.7 (13)
N1—C2—C3—C4	−177.1 (3)	O9—Sn2—C23—C24	−21.1 (5)
C2—C3—C4—C5	−0.8 (6)	C23X—Sn2—C23—C24	61 (5)
C3—C4—C5—C6	−0.4 (6)	C27—Sn2—C23—C24	150.1 (3)
C3—C4—C5—Cl1	178.1 (3)	O9^i^—Sn2—C23—C24	−98.2 (4)
C2—C1—C6—C5	−1.7 (5)	O7—Sn2—C23—C24	68.4 (4)
C2—C1—C6—C7	177.3 (3)	O7X—Sn2—C23—C24	73.9 (7)
C4—C5—C6—C1	1.7 (5)	Sn2—C23—C24—C25	172.0 (4)
Cl1—C5—C6—C1	−176.8 (3)	C23—C24—C25—C26	67.0 (7)
C4—C5—C6—C7	−177.2 (3)	O9—Sn2—C23X—C24X	115 (2)
Cl1—C5—C6—C7	4.3 (5)	C27—Sn2—C23X—C24X	−93 (2)
Sn1—O3—C7—O4	−4.5 (4)	C23—Sn2—C23X—C24X	13 (3)
Sn1—O3—C7—C6	178.4 (2)	O9^i^—Sn2—C23X—C24X	34 (3)
C1—C6—C7—O4	−128.9 (4)	O7—Sn2—C23X—C24X	−160 (3)
C5—C6—C7—O4	50.0 (5)	O7X—Sn2—C23X—C24X	−155 (3)
C1—C6—C7—O3	48.2 (5)	Sn2—C23X—C24X—C25X	−169.8 (19)
C5—C6—C7—O3	−132.9 (4)	C23X—C24X—C25X—C26X	−170 (3)
O9—Sn1—C8—C9	−61.4 (6)	C24X—C25X—C26X—C26X^ii^	42 (10)
C12X—Sn1—C8—C9	137 (5)	O9—Sn2—C27—C28	18.4 (3)
C12—Sn1—C8—C9	138.2 (10)	C23X—Sn2—C27—C28	−133.4 (9)
O3—Sn1—C8—C9	22.8 (6)	C23—Sn2—C27—C28	−152.9 (3)
C8X—Sn1—C8—C9	23.6 (16)	O9^i^—Sn2—C27—C28	96.7 (3)
O8X—Sn1—C8—C9	−142.2 (8)	O7—Sn2—C27—C28	−72.6 (3)
O8—Sn1—C8—C9	−149.3 (5)	O7X—Sn2—C27—C28	−67.5 (6)
Sn1—C8—C9—C10	179.2 (6)	Sn2—C27—C28—C29	−169.1 (3)
C8—C9—C10—C11	−63.2 (10)	C27—C28—C29—C30	−176.7 (4)

Symmetry codes: (i) −*x*+1, −*y*, −*z*+1; (ii) −*x*, −*y*, −*z*+1.

### Hydrogen-bond geometry (Å, °)

**Table:**

Cg1 is the centroid of the C16–C21 ring.

**Table:**

*D*—H···*A*	*D*—H	H···*A*	*D*···*A*	*D*—H···*A*
C13—H13A···Cg1^iii^	0.97	2.92	3.800 (14)	151

Symmetry codes: (iii) −*x*+1, −*y*+1, −*z*.
